# Investigation of Personalized Visual Stimuli via Checkerboard Patterns Using Flickering Circles for SSVEP-Based BCI System †

**DOI:** 10.3390/s25154623

**Published:** 2025-07-25

**Authors:** Nannaphat Siribunyaphat, Natjamee Tohkhwan, Yunyong Punsawad

**Affiliations:** 1 School of Informatics, Walailak University, Nakhon Si Thammarat 80160, Thailand; nannaphat.sir@wu.ac.th (N.S.); natjamee.to@mail.wu.ac.th (N.T.); 2 Informatics Innovative Center of Excellence, Walailak University, Nakhon Si Thammarat 80160, Thailand

**Keywords:** brain–computer interface (BCI), steady-state visual evoked potential (SSVEP), visual flicker pattern, personalized BCI

## Abstract

In this study, we conducted two steady-state visual evoked potential (SSVEP) studies to develop a practical brain–computer interface (BCI) system for communication and control applications. The first study introduces a novel visual stimulus paradigm that combines checkerboard patterns with flickering circles configured in single-, double-, and triple-layer forms. We tested three flickering frequency conditions: a single fundamental frequency, a combination of the fundamental frequency and its harmonics, and a combination of two fundamental frequencies. The second study utilizes personalized visual stimuli to enhance SSVEP responses. SSVEP detection was performed using power spectral density (PSD) analysis by employing Welch’s method and relative PSD to extract SSVEP features. Commands classification was carried out using a proposed decision rule–based algorithm. The results were compared with those of a conventional checkerboard pattern with flickering squares. The experimental findings indicate that single-layer flickering circle patterns exhibit comparable or improved performance when compared with the conventional stimuli, particularly when customized for individual users. Conversely, the multilayer patterns tended to increase visual fatigue. Furthermore, individualized stimuli achieved a classification accuracy of 90.2% in real-time SSVEP-based BCI systems for six-command generation tasks. The personalized visual stimuli can enhance user experience and system performance, thereby supporting the development of a practical SSVEP-based BCI system.

## 1. Introduction

Brain–computer interfaces (BCIs) are a revolutionary technology that enable the fabrication of assistive devices that utilize brain activity signals [1,2,3]. BCIs can be categorized into two main types based on the electrode acquisition method: invasive and non-invasive techniques. Invasive techniques involve placing electrodes directly on the brain tissue using methods such as electrocorticography (ECoG) and intracortical neuron recording [4,5]. These techniques present high spatial resolution signals but require surgical procedures that present the risk of tissue damage and infection [6,7]. However, brain–machine interfaces (BMI) using neural interfaces present considerable potential in medical applications. Non-invasive techniques utilize sensors positioned on or near the scalp, such as electroencephalography (EEG), functional magnetic resonance imaging (fMRI), positron emission tomography (PET), magnetoencephalography (MEG), and functional near-infrared spectroscopy (fNIRS). These methods typically produce signals that are less reliable and more susceptible to noise, thereby requiring sophisticated signal processing for effective usage [8,9]. EEG is significant in BCI research due to its practicality. The 10–20 system places electrodes on the scalp to capture the brain signals. Recent EEG technology advancements [10,11,12] have led to the fabrication of versatile tools for developing BCI systems, which enhances real-world applications.

EEG records two primary types of signals: (1) spontaneous brain potentials obtained from natural behavior. Slow cortical potentials (SCPs) exhibit unique brain activity patterns [13,14]. Additionally, event-related desynchronization/synchronization (ERD/ERS) exhibits power fluctuations in the EEG signals during physical or imagined motor activity [15,16]. (2) Event-related potentials (ERPs), which are elicited by specific external stimuli. The P300 signal, an ERP, appears to be approximately 300 ms after a stimulus and reflects the cognitive response of an individual [17,18,19]. Another category of ERPs includes steady-state visual evoked potentials (SSVEPs), which are marked by EEG responses observed at the same frequency or harmonics as a visual stimulus [20]. SSVEP has emerged as a leading methodology in recent years owing to its high information transfer rate (ITR) and low training requirements, making it suitable for the development of BCI systems [21,22]. SSVEP-based BCI systems can cause eye fatigue due to visual stimulation, which limits their practical use [23,24]. Optimizing stimuli and designing user-friendly interfaces can improve usability. The existing studies conducted on SSVEP-BCI emphasizes the efficiency and user experience by focusing on the stimuli, algorithms, and applications with additional commands. These studies have analyzed the effect of color, shape, light intensity, flickering, and pattern on the performance and comfort. This enhances the accuracy, reduces discomfort [25,26,27,28,29], and balances responsiveness with fatigue [30,31,32,33]. A novel stimulus pattern was employed to improve the classification accuracy and minimize prolonged exposure effects [34,35,36,37]. The number of commands required for complex BCI control mechanisms have also been expanded [38,39,40]. Additionally, the development of classification algorithms improved the command recognition accuracy and system reliability [33,41,42,43,44]. Advancements in SSVEP-based BCI systems further contribute to innovation and practical applications.

SSVEP-based BCI systems are designed to support several users without adjustments; however, their efficiency can be affected by individual differences in the brain signals. Users typically require time to train the system for a proper response, thereby increasing interest in personalized SSVEP-BCI that adapts to the signals of each user, which enhances accuracy and reduces the training time [45,46]. Table 1 presents the previous studies conducted on personalized SSVEP-based BCI systems. Wen et al. [47] conducted an experiment comparing two stimulus formats: an overlooking map and black and white, at frequencies of 8–12 Hz. They evaluated the personal preferences of the users in terms of comfort and feeling when looking at the stimulus. The results indicated that the preference scores were consistent with the characteristics of each stimulus type. Zhang et al. [48] developed a highly customizable algorithm for the SSVEP system. They first performed BETA algorithm testing and then generated an artificial neural network (ANN) model that can adapt to real-time user input, which effectively reduces the signal loss rate and increases the accuracy of SSVEP signal detection. Furthermore, Kondo and Tanaka [49] analyzed the relationship between the stimulus frequency and the magnitude of the SSVEP component that responds to each individual based on the learning FFT technique. Their results indicated that personalizing the stimulus values for each user can increase the data transfer rate when compared with the conventional method without personalized adjustment. Additionally, Kozin et al. [50] designed a light stimulus with an adjustable time delay, enabling frequency changes from 5 to 25 Hz in increments of 1 Hz, to analyze the relationship between the frequency and SNR for each individual. They employed this data to modify the stimulus frequency based on the responses of the user. Despite a slight delay, users responded strongly to the stimulus. For personalized interface design, Na and Tanaka [51] developed a spelling system using LEDs for English letters and Japanese hiragana characters and optimized the computer keyboard interface for each user. This customization significantly improves the spelling efficiency. Jin et al. developed an SSVEP stimulus for a spelling system that supported more than 10 languages. The system interface structure is optimized to respond to various user scenarios and requirements and can be applied to control other devices, highlighting the system’s versatility [52].

In this study, we developed user-adaptive, visually personalized BCIs. Checkerboard flicker patterns that combine geometric symmetry with flickering enhance the visual distinctiveness and improved user attention, particularly under fatigued conditions. However, their performance must be validated in personalized SSVEP-BCI systems. Consequently, we designed a personalized SSVEP-BCI by adapting visual stimuli to the individual neurophysiological responses and analyzing customizable checkerboard flicker patterns via calibration and user profiles. Adjusting the flicker frequency and pattern enhances the SSVEP signal quality and reduces visual fatigue. The main objectives of this study are as follows: (1) The design of a customizable checkerboard flicker stimuli. (2) Implementation of a personalization framework based on the EEG profiles of users. (3) Evaluation of the classification accuracy, information transfer rate (ITR), and user comfort improvements when compared with non-personalized systems and conventional square-pattern stimuli.

## 2. Materials and Methods

**Figure 1:**
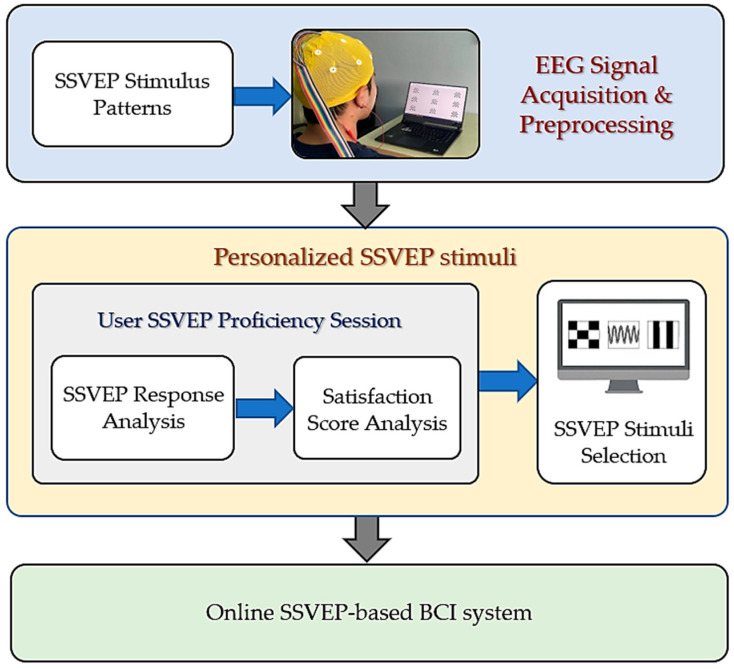
Overview of the proposed personalized visual stimuli for the SSVEP-based BCI system.

In this study, we proposed a system to analyze personalized SSVEP stimuli to enhance the effectiveness of SSVEP-based BCIs. Figure 1 depicts the additional components developed for personalizing the visual stimuli in a typical SSVEP-based BCI framework. Before using the system, the users are required to complete a proficiency session involving various SSVEP stimuli, as defined in the EEG signal acquisition and preprocessing stage. During this session, we analyzed the amplitude and latency of the SSVEP responses and the subjective user satisfaction scores required to identify the most effective individual stimuli. The selected checkerboard flickering circle patterns and EEG channels were employed in an online SSVEP-based BCI system to validate the proposed personalized SSVEP stimuli. Further details are presented in Section 4.

### 2.1. SSVEP Stimuli

Previous studies reported that combining different frequency components in visual stimuli can enhance the strength of the steady-state visual evoked potentials (SSVEPs), demonstrating that individuals may respond differently to high and low frequencies. In our previous study, we developed a novel stimulus pattern to improve the overall response [53]. However, we did not analyze the design of stimuli optimized for individual-specific responses. In this study, we addressed this gap by proposing a personalized approach for SSVEP stimulation. We initially used fundamental frequencies of 7 Hz, 13 Hz, and 17 Hz, along with their first harmonic or subharmonic frequencies, based on both the prior literature and our preliminary investigations [54]. These studies demonstrated reliable neural responses and user comfort within the 6–20 Hz range.

**Figure 2:**
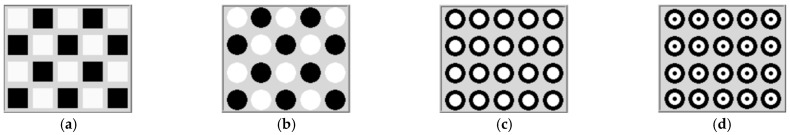
Proposed SSVEP stimuli using checkerboard patterns: (**a**) squares (conventional); (**b**) single-layer circle; (**c**) double-layer circle; and (**d**) triple-layer circle.

The checkerboard pattern with square flickering serves as the conventional stimulus (CB0), as shown in Figure 2a. To analyze the alternative visual configurations, we developed three novel checkerboard patterns using flickering circles: a single-layer flickering circle (CB1), a double-layer flickering circle (CB2), and a triple-layer flickering circle (CB3), as shown in Figure 2b–d, respectively. These were designed with the following considerations: (1) to elicit strong SSVEP responses by varying visual complexity, (2) to minimize visual fatigue by offering patterns with different perceptual loads, (3) to generate more commands by combining different frequency components, and (4) to enable personalization by accounting for individual differences in SSVEP responses.

The CB0–CB3 checkerboard patterns were presented in a fixed sequence to all participants. Each pattern was tested under three frequency conditions: a single fundamental frequency (SFF), the mixing of a fundamental frequency and its harmonic (MFH), and the mixing of two fundamental frequencies (MFF). Table 1 presents the specific frequencies used for each stimulus pattern. This framework was employed to evaluate the participant responses across various patterns and frequency conditions, and to determine the most effective stimulus configuration required for each individual.

The experimental stimuli were arranged in a 3 × 3 grid against a gray background. Each row features distinct frequency compositions: the top row comprises stimuli with single fundamental frequencies (SFF1, SFF2, SFF3), the middle row comprises the mixing of a fundamental frequency with either harmonic or subharmonic frequencies (MFH1, MFH2, MFH3), and the bottom row comprises a mixing of two fundamental frequencies (MFF1, MFF2, and MFF3). The size of each stimulus was 4 × 5 cm, with a spacing of 6 cm between the rows and 12 cm between the columns, designed to minimize visual interference based on our previous study [53], thereby optimizing stimulus discrimination and reducing visual overlap. They were displayed on a 15.6-inch LCD screen with a resolution of 1920 × 1080 pixels.

### 2.2. EEG Acquisition

A total of 15 volunteers (including seven males and eight females, aged 19–22 years) participated in this experiment. All the participants had normal vision, with no color blindness and no history of neurological disorders, including migraines. Informed consent was obtained from all the participants after they were briefed on the experimental procedures. The confidentiality and anonymity of all the participant details were maintained throughout the study. The research protocol was reviewed and approved by the Human Research Ethics Committee of Walailak University (protocol number: WU-EC-IN-2-173-67 on 7 May 2024), corresponding to the ethical principles outlined in the Ethical Declarations of Helsinki, Council for International Organizations of Medical Sciences (CIOMS), and the World Health Organization (WHO).

The Brainmaster Discover 24E EEG machine was employed to measure the EEG data. It features a 24-channel electrode cap with 24-bit resolution and a sampling rate of 256 Hz. A 50 Hz notch filter was employed to remove the power line interference. The recorded signals were processed using a band-pass filter (3–40 Hz) and notch filter at 50 Hz. The EEG signals were collected from 19 channels, including the occipital region electrodes O1 and O2. The A1 and A2 regions comprised the reference electrodes.

### 2.3. Experiments

**Figure 3:**
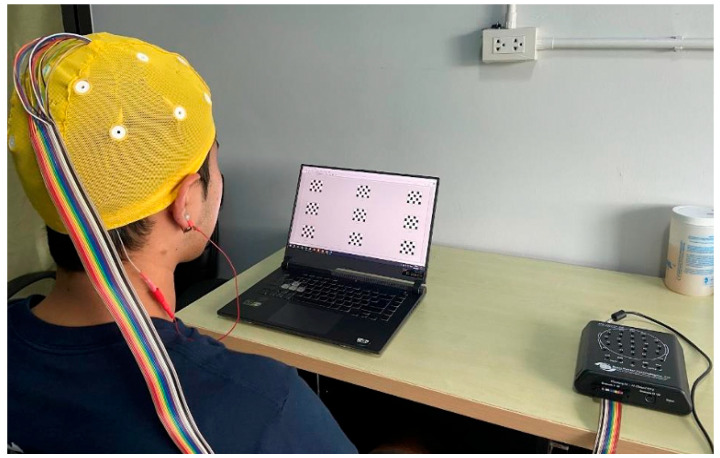
Experiment setup.

In the experiment, the participants sat in a quiet room with standard indoor lighting placed 60 cm away from the computer screen, as shown in Figure 3. All the participants received instructions regarding the experiment. Before the experiment, the participants completed an eye irritation evaluation based on the pain measurement. When participants encountered each stimulus for the first time, they rated their eye irritation level on a scale from 1 to 5. A score of 1 meant comfortable, 2 for rather comfortable, 3 for mildly uncomfortable, 4 for rather uncomfortable, 5 for highly uncomfortable.

**Figure 4:**
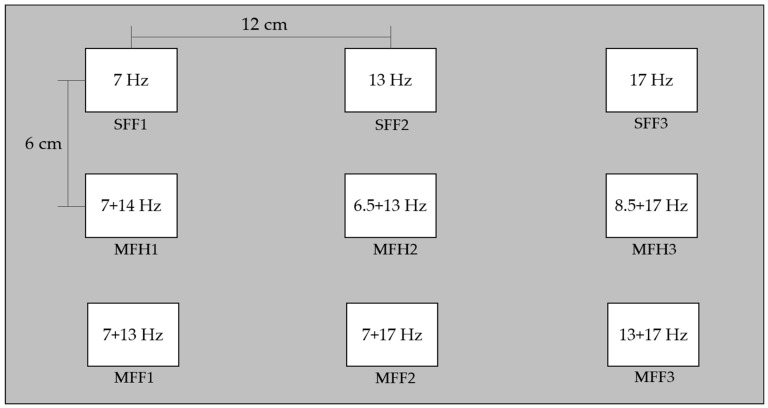
Layout of SSVEP stimuli with three fundamental frequencies and their harmonics for nine different flicker patterns (Hz), as specified in Table 2, displayed on an LCD monitor.

All the visual flicker patterns (Figure 4) were observed simultaneously during the experiments. The participants focused on each flicker in the sequence listed in Table 2, starting with the checkerboard of the flickering squares, followed by three circular flickering patterns, as shown in Figure 2. Each participant completed three sessions for the four checkerboard patterns, with each session comprising 36 trials (four trials per flicker), rest of 5 s, and a stimulus period of 5 s. A 2 min break was provided after each session, along with a 5 min break between the different checkerboard patterns. Each session lasted approximately 6 min.

Furthermore, the participants rated their subjective experience of visual fatigue using a five-point questionnaire after completing each checkerboard pattern. The scale, adapted from a visual analog scale-based pain measurement, comprised five levels of fatigue: 1 (very mild), 2 (mild), 3 (moderate), 4 (high), and 5 (severe). All the participants received standardized instructions on using the scale before the assessment.

### 2.4. Observation of SSVEP Responses from Checkerboard Pattern with Flickering Circles

**Figure 5:**
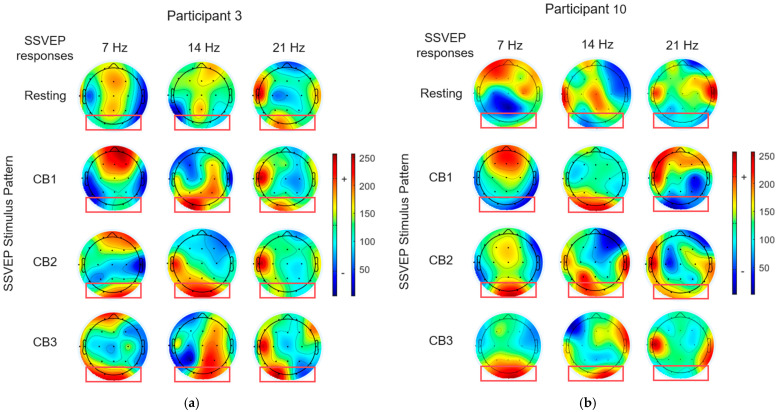
Example of topographic brain maps of SSVEP responses to visual stimulation using the proposed circular checkerboard patterns with a mixture of a fundamental flickering frequency and its first harmonic (MHF: 7 Hz and 14 Hz, respectively). (**a**) SSVEP responses of participant 3 and (**b**) SSVEP responses of participant 10.

**Figure 6:**
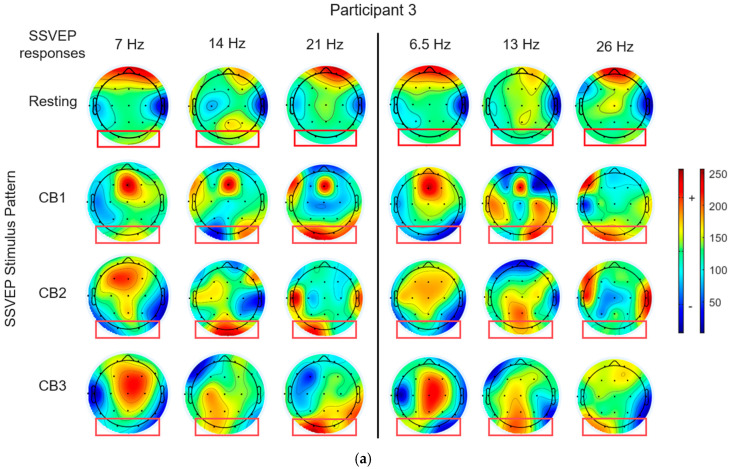

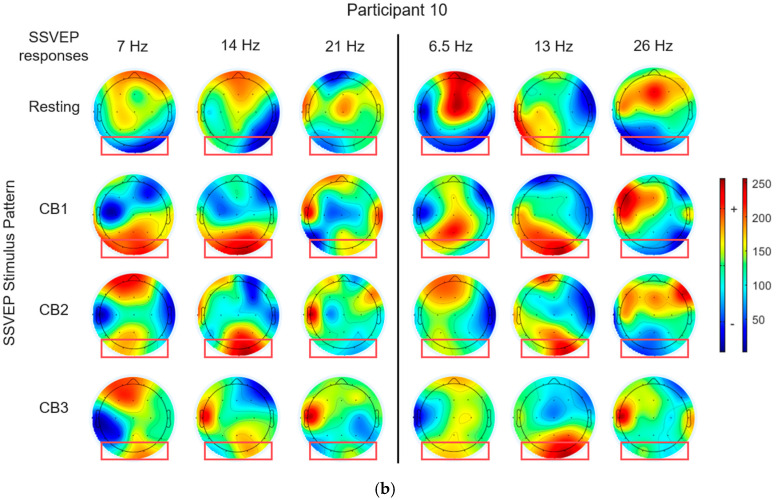
Example of topographic brain maps of SSVEP responses to visual stimulation using the proposed circular checkerboard patterns with a mixture of two fundamental flickering frequencies (MFF: 7 Hz and 13 Hz). (**a**) SSVEP responses of participant 3 and (**b**) SSVEP responses of participant 10.

We employed the EEGLAB Toolbox version 2024.0 [55] to generate topographic brain maps by normalizing the power spectral density (PSD) values to analyze the neural phenomena triggered by visual stimulation, as shown in Figure 5 and Figure 6. The observation focused on the occipital region, particularly at electrode positions, O1 and O2 (indicated by a red box). The color map represents the following: green represents the normalized density PSD, red represents a higher-density PSD, and blue represents a lower density than the normalized density PSD.

Figure 5 depicts the topographic brain maps of the SSVEP responses for participants 3 and 10 (Figure 5a,b) exposed to circular checkerboard patterns (CB1, CB2, and CB3) by combining the fundamental frequency and its harmonic (7 Hz and 14 Hz). When compared with the resting state, all the patterns exhibited strong SSVEP responses at the first harmonic (14 Hz) for both the participants. The CB1 pattern elicited an SSVEP response only at the first harmonic (14 Hz), whereas the CB2 and CB3 patterns elicited responses at both the fundamental frequency (7 Hz) and its first harmonic (14 Hz).

Figure 6 depicts the brain maps obtained from the circular checkerboard patterns at 7 Hz and 13 Hz. The results indicated two distinct frequency components in the SSVEP responses at both the frequencies. Among the three patterns, CB2 produced the strongest SSVEP responses. Higher harmonics (14 Hz and 26 Hz) presented moderate responses, whereas 21 Hz presented a low response. These findings demonstrated that the MFF flicker patterns engage the visual system at target frequencies. Although all the participants exhibited SSVEP responses, their strengths and spatial distributions varied, highlighting the need for personalized visual stimulus designs to enhance the SSVEP-based brain–computer interface (BCI) systems.

The results indicated that brain responses to visual stimuli varied significantly among individuals and were affected by the stimulus pattern and frequency configuration. This variability indicates that the SSVEP responses are specific to each individual. A stimulus that triggered a strong response in one participant may generate a weaker response in another, even when using the same frequency and visual pattern. This observation demonstrates the need for personalized stimuli in SSVEP-based systems using adaptive techniques and user proficiency to optimize the individual performance and enhance the reliability and efficiency of SSVEP signal detection.

### 2.5. Proposed Algorithm

#### 2.5.1. Signal Preprocessing

The signal processing procedures were implemented using MATLAB (MathWorks, version R2020a). A bandpass digital filter with a frequency of 2–40 Hz was applied to reduce power line interference and remove motion artifacts from the EEG signals. We performed signal segmentation using the EEGLAB toolbox. In this study, we initially used a simple SSVEP detection algorithm to verify the proposed stimulus patterns and personalized visual stimuli for SSVEP-based BCI before applying more complex methods.

#### 2.5.2. Power Spectral Density (PSD) and Relative PSD

PSD is a common signal processing technique that illustrates how a signal’s power is distributed across frequencies. EEG signals are transformed from the time domain to the frequency domain, which facilitates the analysis of neural activity related to stimuli, particularly in BCI command decoding. SSVEP responses are characterized by elevated power at the stimulus frequency and its harmonics. We estimated PSD using Welch’s method [20], which segments the signal into overlapping windows and averages the squared magnitudes of the Fourier transforms (FFT), providing a more stable spectral estimate. We also derived relative PSD features by normalizing power at specific frequencies to total control, thereby reducing variability. For SSVEP detection, the relative PSD was calculated as the summed PSD values at the target frequencies and their harmonics divided by the total PSD across all relevant flicker frequencies [56].

#### 2.5.3. Calibrating and Feature Parameters

For feature extraction, we employed the Welch method to estimate the PSD. The relative PSD was calculated as the ratio of the PSD at the fundamental and harmonic frequencies to the total PSD across the frequency band [54]. The EEG data from channels O1 and O2 were used for the SSVEP classification. Based on the results presented in Section 2.4, we proposed an algorithm for individual users for offline (Section 2 and Section 3) and online testing (Section 4). The PSD and relative PSD [56] obtained during the resting state and first trial of each SSVEP stimulation were used for calibration.

The magnitudes (P) of the PSD and the relative PSD at the fundamental flickering frequency ($f_{i}$) and their first and second harmonics. The frequencies of interest were 7, 14, 21, 6.5, 13, 26, 8.5, 17, and 34 Hz.

To account for adjacent frequency influences, the neighboring bins $f_{i-r}$ and $f_{i+r}$ were also considered, where r = $f_{s}/N$, the sampling rate ($f_{s}$) was 256 Hz, signal length was 4 s, and window size (N) was 1024. The calibration and feature extraction processes consisted of the following four steps:

(1) Calibrating: Before using the proposed system, the baseline parameters for each frequency component were determined by identifying the maximum and relative PSD values among the frequency of interest ($f_{i}$) and its adjacent frequencies $f_{i-r}$ and $f_{i+r}$.

Two baseline conditions were calculated for each of the five trials: the resting baseline (${BL}_{R}$) and stimulated baseline (${BL}_{S}$), which can be calculated as follows:

(1) $${BL}_{i}=max\left(P_{\left(f_{i-r}\right)},P_{\left(f_{i}\right)},P_{\left(f_{i+r}\right)}\right)$$

(2) Thresholding: To distinguish between resting and stimulated states, threshold coefficients were determined based on the SSVEP response relative to baseline EEG activity. These thresholds aimed to balance sensitivity and specificity for accurate SSVEP detection and were calculated as follows:

The resting threshold ($T_{R(i)}$) was calculated using Equation (2) to detect the whether an SSVEP response as follows:

(2) $$T_{R(i)}=1.5\;*\;B_{R(i)}$$

A response was considered to be present if the stimulation baseline (${BL}_{S}$) exceeded the resting threshold ($T_{R(i)}$).

The stimulus thresholds ($T_{S(i)(min)}$ and $T_{S(i)(max)}$) were defined to assess whether a frequency bin reflected an active SSV EP target as shown in Equations (3) and (4), respectively:

(3) $$T_{S(i)(min)}=0.75{\;*\;B}_{S\left(i\right)}$$

(4) $$T_{S(i)(max)}=1.25\;*\;B_{S(i)}$$

(3) Feature extraction: For each frequency component, a decision rule was applied to determine whether it belonged to a valid SSVEP response. The SSVEP feature parameters (${Sf}_{i}$) were calculated as follows:

(5) $${Sf}_{i}=\left\{\begin{array}{c} {P_{\left(f_{i}\right)}}_{\;-}\;\;T_{R(i)}\;\;\;\;\;,\;{\;T_{S(i)(max)}<P}_{\left(f_{i}\right)}>T_{S(i)(min)}\;\;\;\\ \;\;\;\;\;\;\;0\;\;\;\;\;\;\;\;\;\;\;\;\;\;\;,{\;T_{S(i)(max)}>P}_{\left(f_{i}\right)}<T_{S(i)(min)} \end{array}\right.$$

For each participant, 288 trials were recorded following the procedure in Section 2.3, using SSVEP features extracted from channels O1 and O2. The trials involved four checkerboard patterns and nine flicker patterns, with 72 trials per checkerboard. Each session consisted of 36 trials, with four trials per flicker pattern. The dataset was organized into a matrix of [288 × 2 × 9], with class labels assigned before element detection.

**Figure 7:**
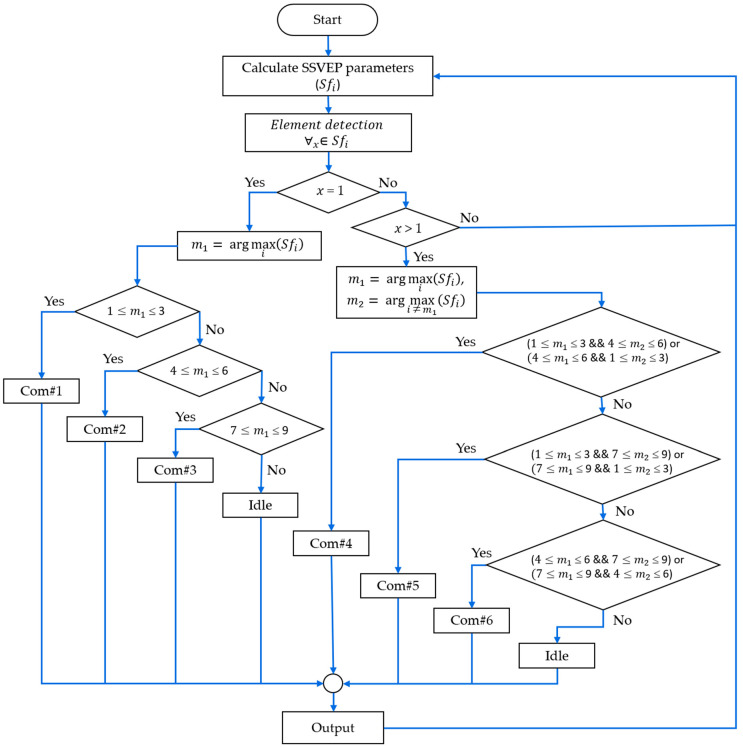
Flowchart of the classification process of the face-machine interface for simulated wheelchair control.

(4) Element detection: After obtaining the parameters, we performed element detection to determine the appropriate SSVEP feature values, as shown in Figure 7. The argmax function was used to identify the peak response within the output parameters.

- For the SFF and MHF patterns, where only one element in the SSVEP response $\left({Sf}_{i}\right)$, is non-zero, the highest value ($m_{1}$) was calculated as follows:

(6) $$m_{1}=\;\arg\underset{i}{\max}\left({Sf}_{i}\right)$$

- For the MFF patterns, where two elements in ${Sf}_{i}$ are of interest, a sorting-based method was employed to obtain the indices of the two largest values. Firstly, the index of the maximum value ($m_{1}$) was identified using Equation (6). Subsequently, the second-highest value ($m_{2}$) was determined by excluding the first maximum and searching for the next, as follows:

(7) $$m_{2}=\;\arg\underset{i\neq m_{1}}{\max}\left({Sf}_{i}\right)$$

#### 2.5.4. Decision Making

After calculating the feature parameters, the system used the values of $m_{1}$ and $m_{2}$, along with the index frequency (i) to classify six commands and an idle state, as shown in Figure 7. For the SFF and MHF flicker patterns, we employed a conventional decision rule using only $m_{1}$ to classify Commands 1 to 3. For the MFF patterns, a two-stage method utilizing both $m_{1}$ and $m_{2}$ was employed to detect two fundamental flickering frequencies, enabling the classification of Commands 4 to 6.

## 3. Results

### 3.1. Evaluation of Checkerboard Pattern Using Flickering Circles

**Figure 8:**
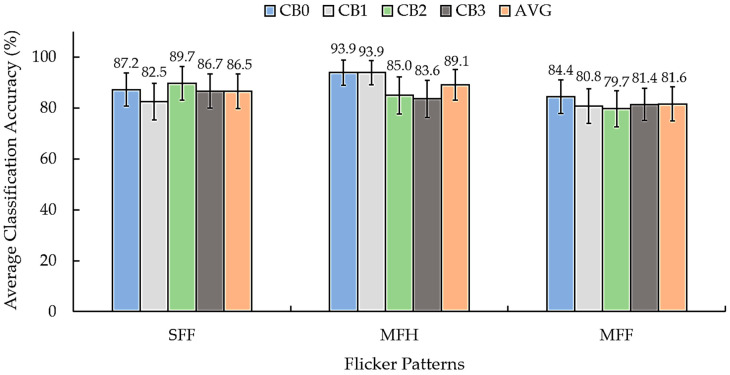
Average classification accuracy (%) across four checkerboard configurations (CB0–CB3) and the overall average (AVG) for each flicker pattern: SFF, MFH, and MFF.

We analyzed two issues with the proposed SSVEP stimuli based on the results presented in Figure 8. Furthermore, we analyzed the two main aspects of the proposed SSVEP stimulus patterns. The first aspect involves evaluating the flicker patterns. MFH achieved the highest average classification accuracy of 89.1% among all the conditions, followed by SFF (86.5%) and MFF (81.6%). MFH consistently outperformed SFF across all the checkerboard patterns, indicating that the inclusion of harmonic frequencies enhances the effectiveness of SSVEP stimulation, which concurs with the previous studies [53]. The second factor is the efficiency of the checkerboard patterns. The average classification accuracy for the conventional CB0 pattern ranged from 84.4% to 93.9% across all the flicker conditions. The CB1 pattern achieved accuracies between 80.8% and 93.9%, whereas those of CB2 ranged from 79.7% to 89.7%. Additionally, CB3 achieved a slightly lower performance, with accuracies ranging from 81.4% to 86.7%. Table 3 presents the detailed classification results for each participant across all the checkerboard and flicker pattern combinations.

The results indicate that the relative PSD method achieved a higher efficiency than the PSD method in terms of the classification accuracy across all three checkerboard patterns (CB1, CB2, and CB3). The highest average accuracy with the relative PSD was observed for CB1 (85.7%), followed closely by CB2 (84.8%) and CB3 (83.9%). This indicates that the relative PSD can more effectively capture the frequency-specific SSVEP features, contributing to accurate classification. The PSD method exhibited a lower performance, with average accuracies ranging from 73.6% to 88.9%. While still viable, the lower accuracy indicates that the PSD may be less sensitive to the transient and harmonic components of the SSVEP responses when compared with the relative PSD. These results show that relative PSD yields more accurate SSVEP classification than conventional PSD under tested flicker and checkerboard conditions. The variation in the individual participant performance (e.g., 75.0% to 97.2% for relative PSD and 73.6% to 88.9% for PSD) demonstrates the inter-subject variability, highlighting the importance of personalized BCI design.

### 3.2. Observation of Eye Irritation and Visual Fatigue

We also observed eye irritation and visual fatigue, which was reported by the participants during the SSVEP stimulus with the proposed checkerboard and flicker patterns. The participants provided feedback on discomfort, which helped in assessing the impact of visual stimuli on eye strain and cognitive overload fatigue to evaluate user comfort and long-term usability.

**Figure 9:**
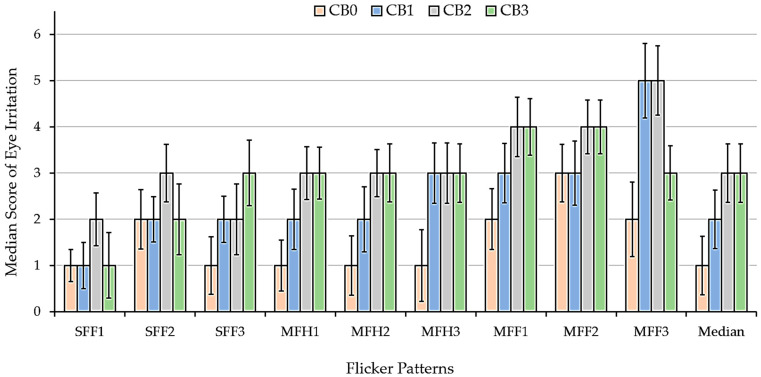
Eye irritation scores from all participants before testing the checkerboard and flicker patterns, with 95% confidence intervals.

As shown in Figure 9, for the checkerboard patterns using flickering circles, the single-layer flickering circle (CB1) induced less eye irritation than the multi-layer flickering circles (CB2 and CB3), as demonstrated by the lower median irritation scores. However, CB1 caused slightly more eye irritation when compared with the conventional flicker square (CB0).

The MFF flicker pattern induced the maximum eye irritation, particularly in MFF3 at high frequency (13 Hz + 17 Hz). The SFF flicker pattern induced minimal eye irritation, particularly at a frequency of 7 Hz. The MFH caused more eye irritation than the SFF flicker pattern. The perspective of the participants indicates that the single circular checkerboard stimulus exerts a marginally greater visual fatigue effect than the conventional checkerboard stimulus. Conversely, the double and triple circular checkerboard stimuli induced significantly higher levels of visual fatigue.

After the experiment, the participants rated their visual fatigue scores for each stimulus (Table 2). The visual fatigue scores were measured on a five-point scale, demonstrating the connection between the flicker patterns and user fatigue, as shown in Table 4. The CB0 pattern exhibited the least fatigue, with a median score of 2 (mild) and a mean score of 2.53, indicating minimal discomfort. CB1 exhibited a slight increase in fatigue, with a median of 3 (moderate) and a mean of 2.80. The transition from square to circular flickering appeared to cause a small increase in the strain. CB2 and CB3 caused significantly more fatigue, with a median score of 5 (severe). The mean scores were 3.87 for CB2 and 4.27 for CB3, respectively, indicating fatigue levels ranging from high to severe, potentially due to the increased flicker density and visual complexity.

The eye irritation and visual fatigue scores indicated user satisfaction with each pattern. We also used irritation and visual fatigue scores to select the visual stimuli for personalized SSVEP visual stimuli.

## 4. Personalized SSVEP-Based BCI via Visual Stimuli

### 4.1. Individual SSVEP Stimulus Patterns

We conducted two stages to identify the optimal visual stimuli for the proposed personalized paradigm (Figure 1), corresponding to the flicker frequencies used to generate the six commands described in Table 5, as outlined by the following algorithm:

Stage 1: The selection was based on notable SSVEP response results (Section 3.1), eye irritation, and the visual fatigue scores (Section 3.2). For each candidate stimulus, a total score was calculated using the formula: Total Score = (classification accuracy − eye irritation score − visual fatigue score). The stimuli with the highest total scores were selected.

Stage 2: If multiple candidates remained, the user preferences were utilized for final selection. The personalized stimulus patterns and corresponding EEG channels summarized in.

**Table 5:** SSVEP stimulus patterns of each frequency pattern for each participant.

Commands	SSVEP Stimuli	
	Flicker Frequencies	Flicker Patterns
1	7 Hz	SFF1/MFH1
2	13 Hz	SFF2/MFH2
3	17 Hz	SFF3/MFH3
4	7 + 13 Hz	MFF1
5	7 + 17 Hz	MFF2
6	13 + 17 Hz	MFF3

The personalized stimulus patterns and corresponding EEG channels for each participant are summarized in Table 6. The combination of CB1 and MFH for Commands 1–3 effectively integrates visual comfort with robust SSVEP responses across participants. CB1 is associated with lower irritation and fatigue levels, whereas MFH elicits strong SSVEP responses.

### 4.2. Real-Time Personalized SSVEP-Based BCI System

**Figure 10:**
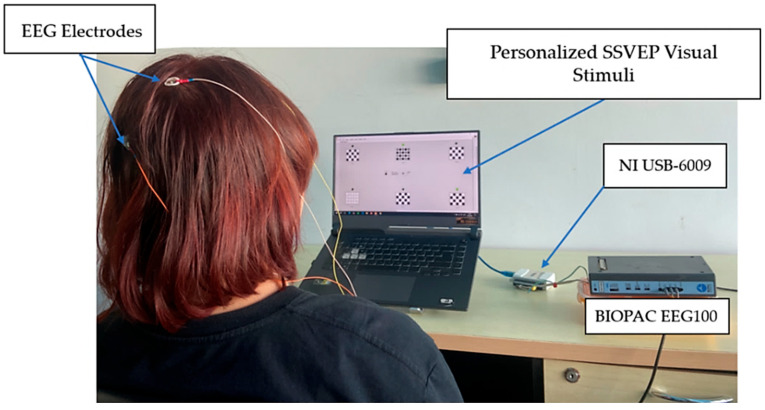
Experimental setup of the proposed personalized SSVEP-based BCI system.

Following the results presented in Section 3, the EEG signals were acquired from a single occipital channel (O1 or O2, as shown in Table 6) using a BIOPAC™ EEG100C amplifier, with the electrode placement based on the international 10–20 system. The signals underwent initial analog filtering using a bandpass filter with a frequency of 1–35 Hz to reduce artifacts and a 50 Hz notch filter to suppress the power line interference. We employed a National Instruments USB-6009 data acquisition device (Austin, TX, USA) to digitize the signals at a sampling rate of 256 Hz. Subsequent digital processing involved applying a bandpass filter with a frequency of 3–35 Hz to remove the noise and motion artifacts. The performance of the system was evaluated by analyzing its ability to accurately classify user-intended commands in real time, as shown in Figure 10.

### 4.3. Evaluation of Personalized SSVEP-Based BCI System

To evaluate the effectiveness of the individual stimuli, we conducted an experiment to compare the mutual stimulus with the preferred stimulus of the subject. For Commands 1 to 3, the mutual stimulus in the CB1 pattern, encoded in the MFH frequency, was compared with the personalized stimulus, which could be either MFH or SFF, based on the individual. For Commands 4 to 6, the mutual stimulus in the CB0 pattern, encoded in the MFF frequency, was compared with the preferred stimulus of the subject encoded in the MFF frequency.

For the experiment, all the participants received training for 15 min to operate the system. Each participant completed three trials for each SSVEP stimulus pattern (mutual and individual). Based on the sequence presented in Table 5, there are six commands; after completing one trial, the participants took a 5 min break before starting the next trial.

Table 7 presents the average classification accuracies for the six comparisons of the mutual and individual SSVEP visual stimulus patterns. For Commands 1–3, the mutual pattern achieved a maximum average accuracy of 93.9%, whereas the individual patterns presented slightly better performance, with an average accuracy of 94.2%. For Commands 4–6, the mutual pattern exhibited an average accuracy of 84.4%, whereas that of the individual patterns reached 86.2%. These results indicate that the proposed personalized visual stimuli method can enhance the classification performance.

However, subject-level analysis revealed that seven participants achieved higher accuracy with the individual pattern for Commands 1–3, while eight participants demonstrated higher accuracy with the individual pattern for Commands 4–6. This suggests that, although individual patterns can enhance overall performance, their benefits may vary among users. Additionally, since the mutual pattern is based on group-level performance, it may not be perfect for every individual. Therefore, additional research is needed to evaluate the long-term effectiveness and user-specific suitability of mutual versus personalized SSVEP stimuli. Therefore, the efficiency of using SSVEP needs evaluation to determine its long-term effectiveness and user-specific suitability of mutual versus personalized stimuli.

Additionally, significant differences were observed when the original stimulus was compared with the preferred stimulus of the subject. The stimulus that combined the fundamental and harmonic frequencies varied significantly from both the original and preferred stimuli (*n* = 15; *p* < 0.05). Similarly, stimuli with two fundamental frequencies also exhibited significant differences from the other two patterns (*n* = 15; *p* < 0.05). However, no significant difference was observed between the fundamental–harmonic and the two-fundamental frequency stimuli (*n* = 30; *p* > 0.05).

## 5. Discussion

### 5.1. Checkerboard Pattern with Flickering Circles

Following the preliminary study presented in Section 2.4, analysis of the proposed checkerboard patterns with flickering circles, and assumption [54], the brain topographic maps revealed SSVEP responses at fundamental and harmonic frequencies, primarily over the occipital regions (O1 or O2). The classification accuracies achieved across the three checkerboard patterns using flickering circles (CB1–CB3) and three flicker patterns (SFF, MHF, and MFF) implemented via the proposed algorithm using the PSD method were comparable to those of the conventional square checkerboard (CB0). When compared with the three checkerboard patterns with flickering circles, the single-layer flickering circle (CB1) achieved the highest accuracy of 93.3% with a mixed fundamental frequency and its harmonic (MFH) flicker pattern using the relative PSD method. The double-layer flickering circle (CB2) achieved the highest accuracy of 89.7% with a single fundamental frequency (MFH). The triple-layer flickering circle (CB3) achieved the highest accuracy of 81.4% with a mixing of two fundamental frequencies (MFF). When compared with the conventional checkerboard pattern (CB0), the CB1 pattern exhibited higher accuracy for both the SFF and MFH flicker patterns. However, all the checkerboard patterns utilizing flickering circles exhibited lower accuracy than CB0 in the case of the MFF. The SFF approach was more effective for the double-layer (CB2) and triple-layer (CB3) flickering circle stimuli, as shown in Figure 7. However, the individual-level analysis presented in Table 3 exhibits variability in the stimulus preferences, demonstrating that personalized stimulus selection can enhance efficiency.

Furthermore, we observed the effects of each stimulus pattern on visual irritation and fatigue. When compared with the conventional stimulus pattern, the CB1 stimulus pattern caused a slight increase in visual irritation and fatigue, whereas the CB2 and CB3 patterns caused a significantly greater visual impact. The visual fatigue and irritation corresponded to the decreased performance in most participants. The multi-layered structures in CB2 and CB3 can increase the visual complexity or cause phase inconsistencies, which may reduce the clarity and strength of the evoked signals and potentially affect visual fatigue and irritation. These findings are consistent with previous research by Ye et al. [57], which demonstrated that visual complexity and display conditions affect visibility and visual comfort. This highlights the importance of considering perceptual factors when designing effective and user-friendly SSVEP stimuli.

### 5.2. Personalized Visual Stimuli for SSVEP-Based BCI

The results presented in Section 3 indicate that the optimal checkerboard and flickering patterns varied across the participants, promoting the development of personalized visual stimuli for the SSVEP-based BCI. We compared the real-time classification accuracy of six commands using mutual and personalized stimuli, which were selected based on the efficiency, subjective visual irritation, and fatigue. The classification algorithm detected the dominant evoked frequencies and the fundamental or harmonic frequencies based on the neural responses of each participant. Overall, personalized stimuli presented consistently higher classification accuracy, demonstrating the effectiveness of individual optimization.

The individual patterns achieved slightly better average accuracies than the mutual patterns: 94.2% and 93.9% for Commands 1–3 using the SFF or MHF flickering pattern, and 86.2% and 84.4% for Commands 4–6 using the MFF. Although the results demonstrated the potential benefits of personalized stimuli, the subject-level analysis indicated that some participants performed better with the mutual pattern. This variability demonstrates the importance of adaptive stimulus selection strategies that consider the individual user responses to maximize the BCI performance. The proposed visual stimulus patterns could be used as alternative SSVEP stimuli by individual users.

### 5.3. Limitations

Despite the promising results, this study has several limitations:

(1) The personalized stimuli were tested with only a checkerboard pattern using three flickering circles.
(2) The experiments were conducted under controlled laboratory conditions with a relatively small sample size of 15 participants, which may impact generalizability.
(3) The proposed system employs low-complexity classification algorithms.
(4) The proposed methods and system did not demonstrate a significant reduction in the visual fatigue and irritation, although they maintained effective SSVEP elicitation to ensure improved performance.

### 5.4. Suggestions and Future Works

(1) The visual stimuli that consider factors such as complexity, color, and size can enhance the personalization and achieve better concurrence with the individual visual sensitivities.
(2) For practical purposes, the feasibility of employing the proposed BCI system for control application in an actual scenario must be verified.
(3) Adaptive algorithms [58] and user profiling can improve real-time tuning, long-term performance, and the use of other visual stimuli such as quick response (QR) code patterns [53].
(4) Future works must incorporate subjective evaluations and objective measures, such as EEG and eye-tracking data, which may inform hardware and software integration to optimize the user visual comfort in SSVEP-based BCI systems based on the related research [59].

## 6. Conclusions

In this study, we introduced a novel stimulus paradigm for SSVEP-based BCI systems by extending the conventional checkerboard design into three circular variants: single, double, and triple-layer patterns. The system successfully encoded six distinct commands by incorporating fundamental frequencies of 7, 13, and 17 Hz, utilizing three flicker patterns: single fundamental, fundamental with harmonics, and dual fundamentals. The SSVEP responses were classified using the PSD method with relative power analysis. The experimental results demonstrated that the proposed stimuli improved the performance when compared with the conventional checkerboard pattern. Real-time testing further validated the usability and effectiveness of individual stimulus methods. Additionally, personalized visual stimuli presented high performance and reduced user variability, demonstrating the importance of individual adaptation in BCI design. These findings support the development of more efficient, user-friendly, and adaptive SSVEP-based BCI systems for individuals with severe physical disabilities.

## Figures and Tables

**Table 1:** Research on personalized visual stimuli for SSVEP-based BCIs.

Author	Proposed Methods	Outputs
Wen et al. (2022) [47]	Personalized preferences for SSVEP stimuli An overlooking map and a black-and-white stimulus at 8–12 Hz frequencies.	The overlooking map performs better than the black-and-white stimulus in terms of accuracy and preference scores
Kondo and Tanaka (2023) [49]	Personalized stimulus frequencies for high-frequency stimulation (56–70 Hz)	Personalized high-frequency stimuli exhibited an accuracy of 87.19% and an ITR of 29.64 bits/min, both exceeding those of the conventional method
Kozin et al. (2023) [50]	Personalized frequency response with time delay A photostimuli with 60 white light LEDs, operating in frequency ranges from 5 to 25 Hz, with increments of 1 Hz	The responses of each subject to the photostimuli are highly individualized The photostimuli can serve as an effective auxiliary tool for studying the level of subjects’ reactions
Na and Tanaka (2024) [51]	Personalized interface design Group of letters on virtual keyboard with different frequency	The consistency between the participant’s intended text and the text determined through brainwave analysis, with an accuracy rate of 80%
Jin et al. (2025) [52]	Dynamic personalized control interface Multitasking user input interface and scenario building through SSVEP combined with big language modeling	Users can enter commands in supported languages. The model creates a corresponding interface based on the input language

**Table 2:** Specific frequencies pattern for each checkerboard pattern.

Visual Flicker Patterns	Sequence	Symbols	Flickering Frequencies (Hz)		
			1st Fundamental	2nd Fundamental	Sub/Harmonics
Single fundamental frequency (SFF)	1	SFF1	7	-	-
	2	SFF2	13	-	-
	3	SFF3	17	-	-
Mixing fundamentals with harmonic frequencies (MFH)	4	MFH1	7	-	14
	5	MFH2	13	-	6.5
	6	MFH3	17	-	8.5
Mixing two fundamental frequencies (MFF)	7	MFF1	7	13	-
	8	MFF2	7	17	-
	9	MFF3	13	17	-

**Table 3:** Classification accuracy across checkerboard patterns using PSD methods.

SSVEP Detection	Average Classification Accuracy (%)					
	Relative PSD Method			PSD Method		
Participants	Checkerboard Patterns			Checkerboard Patterns		
	CB1	CB2	CB3	CB1	CB2	CB3
1	88.9	81.9	80.6	77.8	79.2	75
2	97.2	86.1	90.3	86.1	77.8	83.3
3	80.6	86.1	81.9	83.3	83.3	84.7
4	80.6	77.8	80.6	77.8	76.4	77.8
5	88.9	90.3	88.9	88.9	81.9	87.5
6	86.1	88.9	83.3	81.9	76.4	76.4
7	75.0	80.6	81.9	88.9	83.3	80.6
8	88.9	86.1	83.3	77.8	80.6	77.8
9	83.3	83.3	84.7	79.2	80.6	75.0
10	94.4	93.1	90.3	83.3	79.2	80.6
11	84.7	91.7	84.7	87.5	83.3	83.3
12	83.3	86.1	83.3	76.4	76.4	83.3
13	81.9	77.8	80.6	88.9	81.9	75.0
14	87.5	80.6	81.9	81.9	80.6	79.2
15	81.9	81.9	81.9	81.9	79.2	73.6
Mean ± S.D.	85.7 ± 5.40	84.8 ± 4.63	83.9 ± 3.24	82.8 ± 4.44	80.00 ± 2.49	79.6 ± 4.21

**Table 4:** Participant visual fatigue scores per checkerboard flicker pattern.

Checkerboard Flicker Pattern	Score of Visual Fatigue		
	Range	Median	Mean ± S.D.
CB0: Flickering squares (conventional)	1–4	2	2.53 ± 0.55
CB1: Single-layer flickering circle	1–4	3	2.80 ± 0.56
CB2: Double-layer flickering circle	2–5	5	3.87 ± 0.59
CB3: Triple-layer flickering circle	2–5	5	4.27 ± 0.53

**Table 6:** Optimal stimulus patterns and EEG channels for each frequency pattern and participant.

Participants	EEG Channel	Individual SSVEP Stimulus Patterns			
		Commands: 1–3		Commands: 4–6	
		Checkerboard	Flicker	Checkerboard	Flicker
1	O1	CB1	MFH	CB0	MFF
2	O1	CB0	MFH	CB1	MFF
3	O2	CB2	SFF	CB2	MFF
4	O1	CB3	MFH	CB0	MFF
5	O1	CB1	MFH	CB0	MFF
6	O2	CB1	SFF	CB3	MFF
7	O1	CB0	MFH	CB3	MFF
8	O1	CB0	MFH	CB0	MFF
9	O1	CB1	MFH	CB0	MFF
10	O2	CB2	SFF	CB1	MFF
11	O1	CB0	MFH	CB1	MFF
12	O2	CB1	MFH	CB2	MFF
13	O1	CB3	SFF	CB1	MFF
14	O1	CB2	SFF	CB0	MFF
15	O1	CB1	MFH	CB1	MFF
Mutual	O1	CB1	MFH	CB0	MFF

**Table 7:** Comparison of average online SSVEP detection accuracy between mutual and individual.

Participant	Average Classification Accuracy (%)			
	Commands: 1–3		Commands: 4–6	
	SSVEP Stimulus Patterns		SSVEP Stimulus Patterns	
	Mutual	Individual	Mutual	Individual
1	100.0	88.9	95.8	81.5
2	100.0	88.9	91.7	81.5
3	91.8	92.6	75.0	81.5
4	79.2	96.3	79.7	92.6
5	100.0	100.0	95.8	92.6
6	83.3	90.0	79.7	88.6
7	87.5	91.2	75.0	85.2
8	100.0	88.9	91.7	77.8
9	95.8	92.3	95.8	85.2
10	91.7	94.9	83.3	77.8
11	100.0	97.4	87.5	92.6
12	95.8	94.9	79.7	85.2
13	87.5	100.0	70.8	88.9
14	95.8	100.0	91.7	88.9
15	100.0	96.3	75.0	92.6
Mean ± S.D.	93.9 ± 6.61	94.2 ± 3.99	84.4 ± 8.53	86.2 ± 5.14

## Data Availability

The data presented in this study are available upon request.
